# The evolutionary history of the stearoyl-CoA desaturase gene family in vertebrates

**DOI:** 10.1186/1471-2148-11-132

**Published:** 2011-05-19

**Authors:** L Filipe C Castro, Jonathan M Wilson, Odete Gonçalves, Susana Galante-Oliveira, Eduardo Rocha, Isabel Cunha

**Affiliations:** 1 Interdisciplinary Centre of Marine and Environmental Research (CIIMAR), CIMAR Associate Laboratory, University of Porto (U.Porto), Portugal; 2 Institute of Biomedical Sciences Abel Salazar (ICBAS), University of Porto (U.Porto), Portugal

## Abstract

**Background**

Stearoyl-CoA desaturases (SCDs) are key enzymes involved in *de novo *monounsaturated fatty acid synthesis. They catalyze the desaturation of saturated fatty acyl-CoA substrates at the delta-9 position, generating essential components of phospholipids, triglycerides, cholesterol esters and wax esters. Despite being crucial for interpreting SCDs roles across species, the evolutionary history of the SCD gene family in vertebrates has yet to be elucidated, in particular their isoform diversity, origin and function. This work aims to contribute to this fundamental effort.

**Results**

We show here, through comparative genomics and phylogenetics that the SCD gene family underwent an unexpectedly complex history of duplication and loss events. Paralogy analysis hints that SCD1 and SCD5 genes emerged as part of the whole genome duplications (2R) that occurred at the stem of the vertebrate lineage. The SCD1 gene family expanded in rodents with the parallel loss of SCD5 in the Muridae family. The SCD1 gene expansion is also observed in the Lagomorpha although without the SCD5 loss. In the amphibian *Xenopus tropicalis *we find a single SCD1 gene but not SCD5, though this could be due to genome incompleteness. In the analysed teleost species no SCD5 is found, while the surrounding SCD5-less locus is conserved in comparison to tetrapods. In addition, the teleost SCD1 gene repertoire expanded to two copies as a result of the teleost specific genome duplication (3R). Finally, we describe clear orthologues of SCD1 and SCD5 in the chondrichthian, *Scyliorhinus canicula*, a representative of the oldest extant jawed vertebrate clade. Expression analysis in *S. canicula *shows that whilst SCD1 is ubiquitous, SCD5 is mainly expressed in the brain, a pattern which might indicate an evolutionary conserved function.

**Conclusion**

We conclude that the SCD1 and SCD5 genes emerged as part of the 2R genome duplications. We propose that the evolutionary conserved gene expression between distinct lineages underpins the importance of SCD activity in the brain (and probably the pancreas), in a yet to be defined role. We argue that an expression independent of an external stimulus, such as diet induced activity, emerged as a novel function in vertebrate ancestry allocated to the SCD5 isoform in various tissues (e.g. brain and pancreas), and it was selectively maintained throughout vertebrate evolution.

## Background

**Figure 1:**
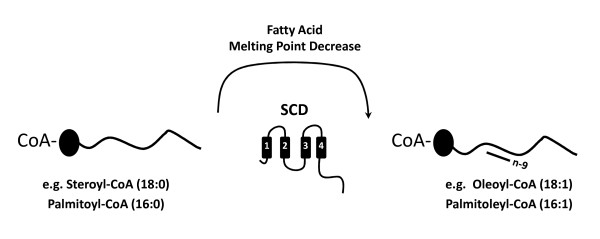
**Stearoyl-CoA desaturase role in fatty acid desaturation**. The four numbered boxes indicate the transmembrane domains.

Fatty acids are chief components of all living organisms, participating in various metabolic processes such as energy storage and as structural elements of biological membranes. They are the components of a wide variety of lipids including oils, waxes, phospholipids and others. Fatty acids occur in saturated and unsaturated forms, a fundamental feature of their physical properties. Diet provides a relevant source of fatty acids. Monounsaturated fatty acid (MUFA) *de novo *synthesis from acyl-CoA involves a series of elongations followed by a final desaturation step. Desaturation and elongation alternating steps, are also key physiological processes in the generation of long chain or very long chain fatty acids (from the essential a-linolenic and linoleic acids). Desaturases comprise a group of membrane-bound enzymes able to activate oxygen and use this reagent to modify C-H bonds at saturated carbons, in diverse substrates such as alkyl groups, acyl residues in thio-, amide- or oxygen-ester linkages, carotenoids, sphingolipids aldehydes and sterols [1]. Their classification into delta-9, delta-6 and delta-5 types reflects the position at which a double bond is introduced counting from the methyl end. In the biosynthetic pathway of MUFAs, a critical committed step is the introduction of the *cis*- double bound in the delta-9 position. This process is catalysed by the steroyl-CoA desaturase (SCD) [2], a rate-limiting enzyme localised to the endoplasmic reticulum and composed of four transmembrane domains (Figure 1). SCDs introduce a double bond at the delta-9 position of saturated fatty acyl-CoAs such as palmitic and stearic acyl-CoA, which are converted to palmitoleoyl-CoA and oleoyl-CoA, respectively (Figure 1). These in turn represent key substrates for the construction of complex lipid molecules such as phospholipids, triglycerides (TG), wax esters, cholesterol esters (CE), and alkyl-2,3-diacylglycerols [3]. Over the years, the accumulated data suggests that SCD expression impacts fatty acid composition of membrane phospholipids, TG and CE, thus changing lipid and lipoprotein metabolism, affecting obesity and membrane fluidity [4]. In fact, fatty acid unsaturation is of paramount importance for the physical properties of membrane lipids. In poikilotherms there is a cold induced increase of membrane lipid unsaturation related to a compensatory increase in the fluidity and conservation of membrane physical properties and functions (homeoviscous adaptation). The ratio of oleic and stearic acid is crucial to the fluidity of membranes and cell-cell interaction [5].

The regulatory setting governing SCDs is largely derived from data of the four tandem linked genes in the mouse. SCD1 is highly expressed in liver and adipose tissue (although ubiquitous in the organism) and has a clear upward trend in high carbohydrate diet fed animals [6]. A downward expression tendency is observed during starvation and with polyunsaturated fatty acid (PUFA) rich diets, with an inhibition of the liver SCD, which is also observed in humans [7-9]. Significantly, SCD1 knockout mice have an attenuation of the obese phenotype when on a high fat diet [10]. There are also some naturally occurring SCD1 deficient mice strains (*asebia *mouse), which, amongst other traits have impaired triglyceride biosynthesis [11]. In fishes, the impact of dietary lipid contents and temperature has also been reported [12]. In the common carp, the genome specific duplication has generated two isoforms expressed in the liver [13]. A decrease in ambient temperature causes a transient upregulation in one isoform, while dietary fatty acid composition modulates the expression of a second isoform [14]. Overall, regulation of SCD gene expression underscores responses to dietary, thermal and hormonal treatment [15].

The delta-9 genes are universally present in living organisms. Surprisingly, we find remarkable variability in the gene complement of SCDs in vertebrate species. The rodent reported gene number varies between four isoforms (SCD1-SCD4) in *Mus musculus *(in a 200 kb span of chromosome 19) and three in *Mesocricetus auratus *(golden hamster) [16,17], though the absence of full genome information in the later precludes conclusions regarding the absence of a forth gene. In humans two genes have been characterised (SCD1 and SCD5), SCD1 being co-orthologous to the four mice genes [13]. SCD5 was initially thought to be primate exclusive, but has now been found in other mammals (but not in the mouse) and birds [18-20]. SCD5 is mostly expressed in the brain and pancreas, in both mammals and chicken [20]. Teleost genes are clearly SCD1-type with no report so far on the presence of SCD5 [13]. On the whole, the current distribution of SCD genes in the various vertebrate species provides no clear evolutionary scenario for the origin of SCD1 and SCD5 (and their function). Two rounds of genome duplication (2R) in vertebrate ancestry have now been firmly demonstrated with the sequencing of the *Branchiostoma floridae *(amphioxus) genome [21]. As a consequence, numerous gene families have specifically expanded up to four copies in the vertebrate lineage. An extra genome duplication in teleosts (3R) [22] contributed further to gene number increase. With respect to the SCD gene family, while the two SCD1 teleost isoforms have clearly resulted from 3R [13], the duplication origin of SCD1 and SCD5 is unclear. In fact, whereas SCD1 is present in both Actinopterygii and Sarcopterygii, SCD5 has only been reported in Amniotes. Thus, it is uncertain whether these genes are ancient paralogues, with their origin coinciding with the appearance of vertebrates; or if they result from a more recent event of gene duplication in the Amniote lineage. Phylogenetics, comparative genomics and examination of evolutionarily informative species should clarify these issues.

The integration of the reported gene diversity with the functional physiological impacts requires the clarification of the SCD evolutionary path. Here, we provide a clear insight into SCD genes in vertebrate history by means of comparative genomics, phylogenetics and gene expression. We determine the timing of the duplication event which gave rise to SCD1 and SCD5 paralogues and propose a scenario for the evolution of gene function in the SCD gene family.

## Results and Discussion

### Human SCD1 and SCD5 map to the NK-linked paralogon

The first human SCD gene to be described, SCD1, localizes to the long arm of chromosome 10. Importantly, this chromosome region is part of the so-called NK homeobox gene paralogon [23,24]. Paralogons are formed by paralogy regions, which in vertebrates largely comprise of a series of unrelated linked genes on one chromosome, which have linked paralogues on three other chromosome regions (or composite regions) due to the 2R events in early vertebrate evolution. The designation of the NK-linked paralogon results from the widespread presence of numerous NK homeobox genes throughout a four composite arrangement, involving chromosomal segments in human chromosomes 2/8 (Hsa2/8), human chromosome 4 (Hsa4), human chromosome 5 (Hsa5), and human chromosome 10 (Hsa10) [23,24]. SCD1 maps in the proximity of a NK cluster gene pair (TLX1/LBX1) at Hsa10 [22]. A second SCD gene, named SCD5, was recently isolated and characterized in humans [18]. It maps to the q arm of Hsa4, a location which is also part of the NK gene cluster paralogon, and close to an NK homeobox gene NKX6.1 (~1.9 Mb). Taken together, both sites are highly indicative of a potential involvement of 2R genome duplications in the origin of these two genes. If correct, this would imply that SCD1 and SCD5 are old paralogues having emerged in vertebrate ancestry as a consequence of 2R.

**Figure 2:**
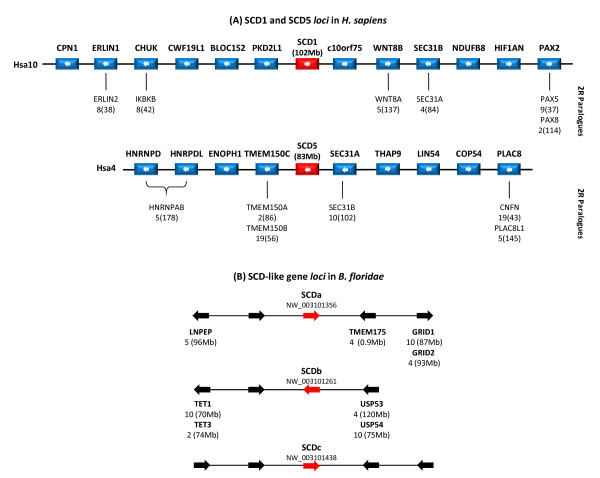
**(A) Chromosomal location of the SCD1 and SCD5 genes in *Homo sapiens*, and their neighbouring genes**. Paralogues of gene families with multiple members are shown below each ORF, with distance in Mb to the p telomere of the respective chromosome. Arrows denote gene orientation. Phylogenetic analysis was performed to unveil their duplication pattern (Additional file_1) unless already available. (B) Genomic locus of *Branchiostoma floridae *SCD-like genes and the neighbouring gene families whose human paralogues localise to expected regions of human SCD paralogy (Hsa10, Hsa4, Hsa5 and Hsa2/8). Gene families with no clear homologues in humans, or gene families that do not localise to the NK-linked paralogon are not labelled (e.g. BfSCDc). The GenBank accession number of each *B. floridae *SCD scaffold is shown.

We analysed in detail the gene family content in the immediate proximity of SCD1 and SCD5 genes in the human genome (Figure 2(A)), to determine their duplication and mapping patterns. We find twelve and nine open reading frames surrounding SCD1 and SCD5, respectively, within a 1 Mb interval (Figure 2(A)). Of the various gene families, the majority are single copies. Of those which are multi-copy, we find that their duplicate paralogues typically localize to regions of the genome included in the NK-linked paralogon (Figure 2(A)). This is the case for WNT8B (Hsa10), which has a second member localising to Hsa5 (an expected region of paralogy) [22]. Phylogenetic analysis of this gene family shows a clear pattern of pre-teleost/tetrapod duplication, in agreement with 2R (additional file 1). Likewise, in the mapping region of SCD5 we also find gene families with duplicated members in expected regions of the NK-paralogon. The HNRNPD/HNRPDL pair has a paralogue mapping to Hsa5 at 4 Mb from the NK-like homeobox gene MSX2. However, the most striking case is represented by the SEC31 gene family, which is composed of two members: SEC31A and SEC31B. SEC31A maps next to SCD5 in Hsa4, while SEC31B is in close proximity to SCD1 at Hsa10. The phylogenetic tree also indicates that, as expected for gene families duplicated from 2R, 31A and 31B genes have duplicated at least before the divergence of teleosts and tetrapods (additional file 1). Overall, the gene families within the SCD genomic regions have a duplication history expected under the 2R genome duplications (additional file 1).

**Figure 3:**
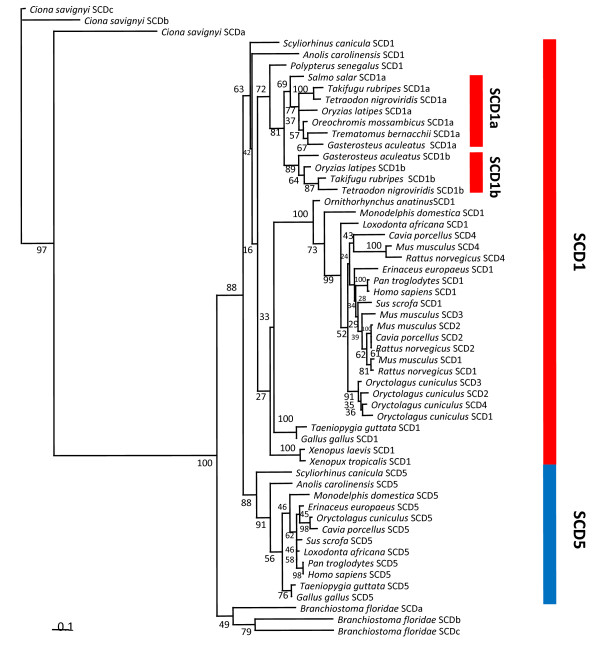
**Maximum likelihood tree of SCD genes, with bootstrap values shown at each node**. *Ciona savignyi *(SCDa [ENSCSAVP00000003593], SCDb [ENSCSAVP00000004039], and SCDc [ENSCSAVP00000003934]), *Branchiostoma floridae *(SCDa [XP_002588865], SCDb [XP_002585987], and SCDc [XP_002596094]), *Scyliorhinus canicula *(ScSCD1 [JF729408] and ScSCD5 [JF729409]), *Anolis carolinensis *(SCD1 [ENSACAP00000010645] and SCD5 [ENSACAP00000010271]), *Polypterus senegalus *(SCD1 [JF729410]), *Salmo salar *(SCD1a [NP_001133452]), *Takifugu rubripes *(SCD1a [ENSTRUP00000031286] and SCD1b [AAU89872]), *Tetraodon nigroviridis *(SCD1a [ENSTNIP00000022600] and SCD1b [ENSTNIP00000002814]), *Oryzias latipes *(SCD1a [ENSORLP00000011565] and SCD1b [ENSORLP00000008592]), *Gasterosteus aculeatus *(SCD1a [ENSGACP00000003149] and SCD1b [ENSGACP00000011262]), *Oreochromis mossambicus *(SCD1a [AAN77732]), *Trematomus bernacchii *(SCD1a [ACI16378]), *O. anatinus *(SCD1 [ENSOANP00000003925]), *Monodelphis domestica *(SCD1 [ENSMODP00000013317] and SCD5 [ENSMODP00000015016]), *Loxodonta african*a (SCD1 [ENSLAFP00000014914] and SCD5 [ENSLAFP00000013895]), *Erinaceus europaeus *(SCD1 [ENSEEUP00000005718] and SCD5 [ENSEEUP00000010623]), *Pan troglodytes *(SCD1 [ENSPTRP00000005002] and SCD5 [ENSPTRP00000027870]), *Sus scrofa *(SCD1 [ENSSSCP00000011244] and SCD5 [ENSSSCP00000009859]) *Homo Sapiens *(SCD1 [ENSP00000359380] and SCD5 [ENSP00000316329]), *Taeniopygia guttata *(SCD1 [ENSTGUP00000007924 and SCD5 [ENSTGUP00000002919), *Gallus gallus (*SCD1 [ENSGALP00000039331] and SCD5 [ENSGALP00000018194]), *Xenopus laevis *(SCD1 [AAH81254]) *Xenopus tropicalis *(SCD1 [ENSXETP00000051240]), *Mus musculus *(SCD1 [CAJ18540], SCD2 [NP_033154], SCD3 [NP_077770] and SCD4 [AAH38322]), *Rattus norvegicus *(SCD1 [NP_631931], SCD2 [NP_114029] and SCD4 [XP_574671]), *Oryctolagus cuniculus *(SCD1 [XP_002718695], SCD2 [XP_002718696], SCD3 [XP_002718697], SCD4 [XP_002718662] and SCD5 [ENSOCUP00000005142]) and *Cavia porcellus *(SCD2 [ENSCPOP00000008837], SCD4 [ENSCPOP00000009812] and SCD5 [ENSCPOP00000002936]).

The localization of the human SCD gene isoforms in a 2R-generated paralogon implies two testable predictions. Unless independent gene expansions have taken place, invertebrate chordates should have a single SCD gene equally related to their vertebrate counterparts. Second, invertebrate SCD genes should be flanked by gene families that have their human orthologues/paralogues localising to regions of SCD paralogy (Hsa4, Hsa10, Hsa5 and Hsa2/8), even if conserved micro-synteny (conservation of immediately adjacent neighbours) is not observed. To test these predictions, we have analysed the genomic *locus *of the SCD gene in the invertebrate chordate *B. floridae*. Although we expected to find a single SCD isoform, our search retrieved three distinct SCD-like genes. Nevertheless, these represent an independent gene expansion in the amphioxus lineage (Figure 3), since they group together outside of the vertebrate SCD1/SCD5 clade (Figure 3). We name these genes BfSCDa, BfSCDb and BfSCDc. With the exception of BfSCDc, we find the amphioxus SCD's flanked by gene families which have their human homologues mapping to regions of SCD paralogy (Hsa4, Hsa10, Hsa5 and Hsa2/8) (Figure 2(B)). For example, close to BfSCDb a single TET1/3 gene is found with the human paralogues TET1 and TET3 mapping to chromosomes 10 and 2, respectively (Figure 2(B)). A similar situation is found close to BfSCDa where a GRID1/2 gene is found. Human GRID1 and GRID2 localise to Hsa10 and Hsa4 as expected, under the scenario that these regions represent an amphioxus-specific partitioning of the 2R unduplicated genomic locus. Thus, although a conserved proximal synteny is not observed, the amphioxus data clearly supports the hypothesis that vertebrate SCD1 and SCD5 expanded in the vertebrate lineage as part of 2R genome duplications.

Upon its initial characterization, SCD5 was proposed to be primate specific [18], while SCD1 had a more widespread presence in vertebrate genomes. However, the more recent identification of SCD5 orthologues in bovines [19], and chicken [20] suggested that the original SCD1/SCD5 duplication pre-dated the timing of the bird/reptile-mammal divergence. Our analysis shows that the genomic location of SCD1/SCD5 and the phylogenetics of flanking gene families indeed supports an older age for the duplication event that gave rise to the SCD1 and SCD5 paralogues. The reported scenario strongly hints that SCD1 and SCD5 are the remaining two duplicates of the four that originated from an ancestral invertebrate SCD gene as a result of 2R. Alternatively, after 1R, one of the duplicated SCD genes was lost leaving one remaining for duplication during 2R, producing SCD1 and SCD5.

### Gene loss and tandem duplications illustrate the tetrapod SCD repertoire

The evolutionary setting emerging from the paralogy analysis creates some important repercussions. For example, the phylogenetic distribution of both SCD isoforms is potentially broader than previously found. Also, the absence of SCD genes (either 1 or 5) in vertebrate classes would mean gene loss and not a different timing of the SCD1/SCD5 gene duplication.

To elucidate these matters, we started by analysing tetrapod species using two strategies. Firstly, by determining the duplication timing through phylogenetics, and secondly by investigating the SCD gene *loci *in available tetrapod genomes representing various lineages. The information on gene complement and *loci *organization was collected from *Homo sapiens *(Placental Mammal, human), *M. musculus *(Placental Mammal, Rodent, mouse), *Ornithorhynchus anatinus *(Monotremata, platypus), *Monodelphis domestica *(Marsupial, opossum), *Gallus gallus *(Bird, chicken), *Anolis carolinensis *(Reptile, anole) and *X. tropicalis *(Amphibian, african clawed frog). The analysis was expanded to include *Oryctolagus cuniculus *(Placental Mammal, Lagomorpha, rabbit) given the surprising number of SCD1 genes.

The orthology of the designated tetrapod SCD1 and SCD5 genes was assessed by the phylogenetic analysis (Figure 3). Although some nodes are poorly supported in the SCD1 branches, in general we find good statistical support for the separation of these two gene lineages, SCD1 and SCD5, in tetrapod species. Within the SCD1 mammalian clade, we find that SCD1-type gene expansion is not restricted to the Muridae family represented here by *M. musculus *and *Rattus norvegicus *(brown rat), though in the later only three SCD genes are found. This increase is extended to the entire Rodentia order for which genome data (partial) is available (not shown). Another mammalian species, *O. cuniculus*, was also found to have an expansion of the SCD1-type sequences. The common ancestry between rodents and lagomorphs probably indicates that the expansion of SCD1 genes took place in the ancestor of both lineages at 83 mya [25]. However, we find the four rabbit genes to cluster strongly together and not with the previously reported *M. musculus *genes (Figure 3). Though this could imply two separate duplication events, we find gene conversion in the rabbit SCD gene cluster a more plausible alternative. As more SCD sequences become available in these groups, a detailed analysis should clarify the origin and functional consequences of the reported phylogenetic pattern.

SCD5 internal nodes are more robustly supported. Within tetrapods we find no SCD5-like sequence in the amphibian *X. tropicalis*. In contrast to mice, *Cavia porcellus *(guinea pig, Rodent) and *O. cuniculus *have SCD5 orthologues (Figure 3).

**Figure 4:**
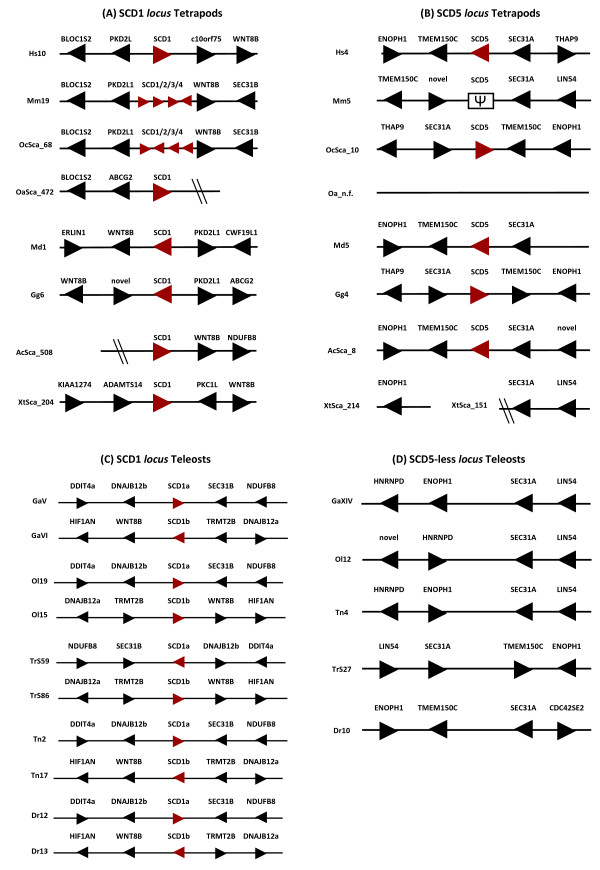
**SCD1 (A), SCD5 (B) gene *loci *in tetrapod species; SCD1a/SCD1b (C) and SCD5 (D) gene *loci *in teleost species**. Hs - *H. sapiens*, Mm - *M. musculus*, Oc - *O. cuniculus*, Oa - *O. anatinus*, Md - *M. domestica*, Gg - *G. gallus*, Ac - *A. carolinensis*, Xt - *X. tropicalis*, Ga - *G. aculeatus*, Ol - *O. latipes*, Tr - *T. rubripes*, Tn - *T. nigroviridis*, and Dr - *D. rerio *Arrow head indicates gene orientation, and oblique lines denote scaffold ending. Ψ indicates pseudogene.

The unsupported position of the amphibian and bird SCD1 genes raises some questions regarding their orthology. Thus, we next inspected the *loci *gene content as a means to determine a common evolutionary origin. The SCD1 gene *locus *is organised in a highly conserved arrangement (Figure 4(A)). Notwithstanding some gene order variation, we find PKD2L1 to outflank SCD1 in most of the analysed species, while WNT8B is typically downstream of SCD1 (Figure 4(A)). We confirm that the mouse SCD1 repertoire is expanded to four members organized in tandem in agreement with previous reports (Figure 4(A)), and similar to what is found in *O. cuniculus *(Figure 4(A)).

Next, we examined the SCD5 genomic *locus *in the various tetrapod species (Figure 4(B)). In contrast to SCD1, no evidence of gene tandem expansion was observed. SCD5 retains a single copy status in a highly conserved arrangement across species (Figure 4(B)). No orthologue was found in *X. tropicalis *or *O. anatinus*. As previously noted SCD5 is absent from *M. musculus *and *R. norvegicus *[20]. However, we find SCD5 orthologues in another Rodentia species (*C. porcellus*), which indicates that the loss of SCD5 is secondary and probably restricted to the Muridae family (Figure 3). In *O. anatinus *and *X. tropicalis*, no SCD5 was identified. However, we suspect that the low coverage of the genome sequence in the case of the platypus is the likely cause for this absence since we were also unable to find other neighbouring genes. In the case of *X. tropicalis*, we find that the genes typically flanking SCD5 in other tetrapod species are present but in different scaffolds (Figure 4(B)). We find the SEC31A gene located at the end of scaffold_151, a possible indication of a sequence gap in the genome assembly. To investigate whether a potential SCD5 sequence was present but not assembled, we searched the trace sequence genome archives. No sequence reads with similarity to SCD5 were found.

An alternate hypothesis, which opposes the paralogy findings, postulates that SCD1/SCD5 isoforms emerged after the divergence of amphibians in the Amniote clade. In that case, the amphibian SCD gene should out-branch the mammalian, bird and reptile SCD1/SCD5 genes in phylogenetic trees. However, we find that the *Xenopus *gene groups with the described SCD1 genes (Figure 3), though the bootstrap value is relatively low (63), when more basal vertebrate lineages are included). Documenting gene loss is a challenging task in the presence of incomplete genomes. Thus, verifying if SCD5 absence is a true biological reality in a second amphibian should address both the technical and general implications of this preliminary finding.

### Teleosts have lost SCD5 and SCD1a/SCD1b are 3R paralogues

The SCD gene complement is unexpectedly variable in tetrapods. Thus, we next inspected various teleost species. These included species with full genomes available such as *Danio rerio *(zebrafish), *Gasterosteus aculeatus *(three-spined stickleback), *Tetraodon nigroviridis *(green spotted puffer), *Takifugu rubripes *(Japanese pufferfish) and *Oryzias latipes *(medaka). Typically, two SCD1 genes have been described in this group [13]. Here, we use a distinct nomenclature for the fish isoforms, SCD1a/SCD1b in contrast to SCD1/SCD2, respectively [13]. Phylogenetic analysis shows that both isoforms are SCD1 and specifically duplicated in the teleost lineage with two strongly supported groups: 1a and 1b (Figure 3) [13]. The *D. rerio *sequences introduced a different topology, in a pattern previously noted [13], and were left out of the phylogenetic analysis. However, the synteny analysis clearly supports that the *D. rerio *genes are also of the 1a/1b type.

The teleost genome has experienced an extra genome duplication (3R), to the two rounds that initially occurred in stem vertebrate evolution [22]. Thus, it has already been shown that the SCD1a/1b duplicates are the result of the 3R [13]. The analysis of the regions harbouring SCD1a/SCD1b in the five species with fully sequenced genomes confirms this pattern (Figure 4(C)) [13]. We find the gene families in the immediate proximity of SCD1a to have duplicate members in the scaffold or chromosome where SCD1b resides (Figure 4(C)). For example, DNAJB12b maps a few kb away from SCD1a, while DNAJB12a is immediately localized downstream of SCD1b (Figure 4(C)).

To demonstrate further that the 1a/1b duplication resulted from the 3R genome duplication, we decided to investigate the basal ray-finned fish, *Polypterus senegalus *(bichir). This basal Actinopterygii diverged pre-3R [26]. Our degenerate PCR approach isolated a single partial sequence (711bp). The inclusion of the bichir sequence in the phylogenetic analysis shows that the *P. senegalus *SCD1 outgroups the 1a and 1b teleost genes (bootstrap 72), adding further support to the proposal that indeed the SCD1a/1b duplication resulted from 3R.

No SCD5 gene sequence has been so far described in teleosts. Furthermore, in available full genome teleost sequences no SCD5 annotation is found. To test whether the absence of SCD5 orthologues was due to genome incompleteness or absence of gene description, we examined the location of the tetrapod SCD5-flanking genes in the available teleost genomes (Figure 4(D)). The similarity in gene arrangement between the fish group and SCD5 tetrapod *loci *is remarkable, and suggests that SCD5 was a targeted deletion in teleosts. We were unable to confirm if this loss event post or pre dates 3R, since our attempts to isolate SCD5 from *Polypterus *were unsuccessful. Thus, it is unclear if the gene complement in *P. senegalus *is restricted to SCD1. In summary, despite the absence of SCD5, we find that teleosts retain an SCD gene complement similar to most tetrapods due to the specific duplication of SCD1 genes.

### SCD1 and SCD5 orthologues are present in the cartilaginous fish *Scyliorhinus canicula*

One of the predictions emanating from the paralogy analysis is that clear SCD1 and SCD5 orthologues should be found in extant vertebrate classes, unless loss events have taken place. SCD5 genes were found in reptiles and birds so far, but not in teleosts with strong evidence for gene loss. To determine whether SCD1 and SCD5 have been preserved in the oldest group of jawed vertebrates, we searched the emerging genome sequence of the elephant shark *Callorhinchus milii *[27]. Also, using a degenerate PCR strategy we aimed at isolating orthologues of the SCD gene family from the lesser spotted dogfish, *S. canicula*. We found partial sequences with similarity to either SCD1 or SCD5 in the elephant shark, but too small to provide solid confirmation (not shown). In the case of *S. canicula*, our approach yielded two distinct sequences with similarity to either SCD1 or SCD5. Sequence extension was achieved with various PCR strategies, resulting in two sequences coding for proteins with 341 and 325 amino acids when finally isolated.

To provide a clear confirmation, we have examined the phylogenetic relationships of the *S. canicula *sequences (Figure 3). We find that these sequences group consistently; ScSCD1 with the SCD1 clade (bootstrap 63), and ScSCD5 with SCD5 genes (bootstrap 88).

### SCD tissue expression suggests the conservation of an ancestral function

**Figure 5:**
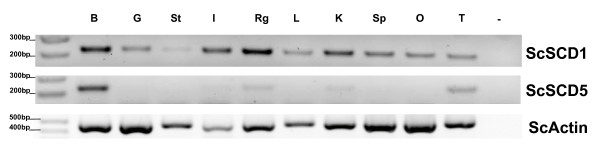
**Gene expression of SCD1 and SCD5 *S. canicula***. B - brain, G-gill, St - stomach, I-intestine, Rg-rectal gland, L-liver, K-kidney, Sp - spleen, O-ovary, and T-testis.

We next addressed the expression location of newly isolated SCD shark genes, as a proxy for functional appraisal. SCD1 genes are ubiquitously expressed in the various species analysed so far, with high expression being observed in the liver and adipose tissue [20]. In contrast, available data on SCD5 shows that in birds and mammals, a distinct localised expression is observed, with emphasis in the brain and the pancreas [20]. In accordance, we find ScSCD1 expressed at variable levels in all the tested tissues (Figure 5), while ScSCD5 has a clear expression in the brain, and a minor expression in the testis and salt secreting rectal gland (Figure 5). A second site of conserved expression between birds and mammals is the pancreas. However, we did not examine the pancreatic expression in *S. canicula*.

**Figure 6:**
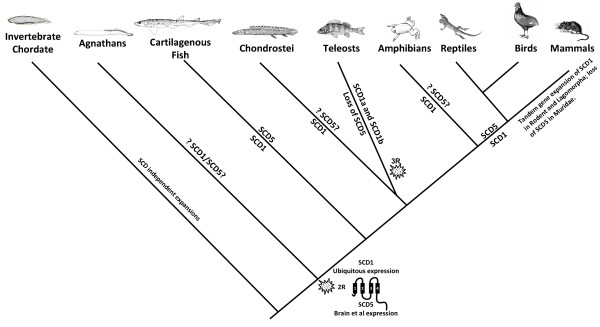
**Proposed evolutionary model of SCD genes in vertebrates**.

At the start of this investigation, we found that paralogy studies suggested a much earlier date for SCD1/SCD5 duplication, probably to the stem of vertebrate evolution. The finding of clear of SCD1 and SCD5 orthologues in a basal jawed vertebrate confirms this hypothesis. The overall phylogenetic analysis also provides insight into a more revealing evolutionary pattern (Figure 6). We note that SCD1 is retained in all lineages examined, with occasional gene expansion either through tandem duplications or as a result of genome duplications. In rodents and lagomorphs tandem duplications have increased the gene number up to three/four members, while in teleosts the 3R genome duplication is accountable for the presence of two SCD1 genes. SCD5 has a more scattered phylogenetic distribution. It has been lost in teleosts and the mammalian Muridae family, with its absence in *X. tropicalis *being impossible to confirm unequivocally at this stage. We conclude that clear SCD1 and SCD5 orthologues were present in basal jawed vertebrate, and loss and duplication events took place during vertebrate evolution (Figure 6).

The parallel expression profile between Amniote (mammal and bird) and Chondrichthyes SCD orthologues, is a remarkable finding in lineages that have been separated for more than 350 million years. It supports some important propositions namely (a) that they represent an ancestraly conserved trait acquired in vertebrates following 2R (e.g. SCD5 brain expression and SCD1 ubiquitous role) and (b) that a selective force must be operating to maintain the proteins with similar roles in these distinct lineages. Paradoxically, we find an evolutionary pattern of clear SCD5 gene loss in at least two groups, which at face value contradict the relevance of the conserved function of SCD1 and SCD5 (Figure 6). For example, we find loss of SCD5 in teleosts and some rodents (Figure 6). This opposes the specific SCD5 localised brain expression as being evolutionarily relevant, unless particular adaptations have taken place. In support, we find some observations to explain partially this apparent contradiction. In mice although having lost SCD5, the SCD1 complement expanded to four genes. The expression profile of the murine SCD1/2/3/4 genes shows a compartmented profile: SCD1 is highly expressed in the liver, SCD4 is exclusively found in the heart, SCD3 is unique to the skin, and SCD2 is almost entirely restricted to the brain [16,28]. Thus, having lost SCD5 and its localized expression in the brain, an apparent functional recruitment of the murine SCD2 has taken place. As for teleosts a more complex outline emerges. While no SCD5 orthologue exists, SCD1b was found to be expressed almost uniquely in the *D. rerio *brain (although in a small tissue panel) [13]. This observation also suggests that a selective pressure is present for the maintenance of a SCD "*brain function*" in zebrafish. This is a noteworthy pattern of apparent functional interchange between 2R and 3R paralogues and echoes the functional swapping between 2R paralogues [29]. The case of the SCD1 in teleosts mimics that of the Cdx transcription factor gene family. The Cdx gene complement is composed of three genes in vertebrate classes (Cdx1, Cdx2, and Cdx4). However, in teleosts Cdx2 has been lost, while Cdx1 has duplicated as a result of 3R [30]. The characterization of Cdx1a/Cdx1b in zebrafish shows that Cdx1b replaced the role of mammalian Cdx2 in gut development [31], in a functionally equivalence process.

To infer whether the teleost SCD1a/b expression profile corresponds to a transversal pattern retained in teleost species needs clarification. Currently, with the exception of zebrafish no other complete dataset involving the expression of both isoforms is available. Thus, we analysed the 1a/1b profile in the stickleback. Surprisingly, in *G. aculeatus*, SCD1a and SCD1b are found ubiquitously (additional file 2), in sharp contrast to the zebrafish. SCD regulation parallels other gene involved in teleost lipid metabolism. ELOV2, an enzyme essential for the elongation of LC-PUFAs, is present in freshwater Ostariophysan species (e.g. *D. rerio*), while absent in the Acanthopterygii order (e.g. *G. aculeatus*) [32]. It has been proposed that the retention of ELOV2 is related with the poor-LC-PUFAs in freshwater habitats, when compared to marine ecosystems which are fuelled with phytoplankton [32]. In the context of the delta-9 desaturation, a distinct regulation of SCD expression in zebrafish and stickleback could be related with distinct availabilities of fatty acids (e.g. oleic acid) in their habitat or differences in feeding strategies. Also, in the common carp two SCD1 genes are expressed exclusively in the liver and subjected to distinct regulation, one of which is independent of dietary inputs [14]. However, these are not directly comparable since they are carp genome specific SCD1a duplicates [13]. We note also that in the grass carp (no genome duplication) SCD1a is expressed strongly in the liver with vestigial signs in the brain [33], but so far no orthologue of SCD1b has been described. Determining the expression of SCD1a and SCD1b genes in a larger panel of teleost species should prove clarifying.

Whereas the function and regulation of SCD1 is widely studied, SCD5's particular function is largely unknown. However, in humans, both genes shown similar subcellular localization [18]. Surprisingly, they also show similar desaturase activities *in vitro*, both producing oleic acid [18]. Where they differ distinctly is in their expression pattern, an aspect which we now conclude dates back to the origin of gnathostomes. The brain SCD5 profile is most suggestive. Oleic acid, one of the outputs of the SCD enzymatic action, is a relevant component of the brain, in particular of myelin [34]. Lengi and Corl [19] have put forward that the maintenance of optimum levels of oleic acid in the brain might signal the potential role of SCD5. In fact, in rats the levels and origin of oleic acid in the brain appear to be independent of the diet [34,35]. In this context and in contrast to SCD1, SCD5 regulation (and function) would be independent (or at least less dependent) of low dietary intake. In agreement, the mouse SCD2 regulation (a potential functional equivalent of SCD5) is not influenced in the adult irrespective of the dietary intake of PUFAs [36]. Thus, we propose that a major distinction between SCD5 and SCD1 would be at the regulatory level, with SCD5 being unresponsive to external inputs (e.g. food sources) in its action in the brain (but probably also other tissues). In this context, the acquisition of this novel setup would allow the maintenance of optimal oleic acid levels in brain cell membranes (an aspect fundamental for its activity), irrespective of environmental availability of lipid food sources. In support, we find that one of the common carp SCD1 specific duplicates behaves independently of dietary variation in the response to cold induction [14].

SCD5 has also been shown to be highly expressed in the pancreas, in birds and mammals [20], a tissue we have not examined in *S. canicula*. Elevated levels of saturated fatty acids have been linked with beta cell death *in vitro *[37]. SCD activity was recently shown to be involved in a cytoprotective mechanism by transferring saturated fatty acids into MUFAs which are then incorporated into lipids [38]. The contribution of SCD5 to this role has not yet been elucidated, as well as its expression pattern under different dietary conditions. To understand if this is also conserved between the various vertebrate species remains a challenge.

Overall, two alternate possibilities can explain the phylogenetics and genomics data reported here. Either, an ancestral SCD gene gave rise to four genes as a result of 2R, with two genes being lost; or after 1R, one SCD gene was deleted with the remaining SCD duplicating during 2R. Both proposed scenarios resulted in the retention of SCD1 and SCD5 in most lineages. We suggest that these genes have diverged functionally, with SCD5 gaining a distinct regulatory program from that of SCD1, probably independent of external dietary lipid inputs.

## Conclusion

We find that the duplication which gave origin to the SCD1/SCD5 paralogues dates back to the 2R genome duplications. Concomitantly, we find SCD1 and SCD5 in a basal jawed vertebrate, the cartilaginous fish. We argue that a regulatory divergence between SCD1 and SCD5, accounts for their functional differentiation in the aftermath of 2R. Thus, a tissue specific expression (e.g. brain and probably pancreas) emerged in vertebrate ancestry allocated to the SCD5 isoform, which has been selectively kept in most lineages. However, exceptions have survived the loss of SCD5. A "brain" specific expression is still retained, albeit through the use of diverse strategies. In muridae, a SCD1-type gene (SCD2) has fulfilled the function. In the teleost *D. rerio*, a 3R SCD1 gene also has a localised expression in the brain. We consider that the interplay of gene regulatory setups in a selective environment was at play in the SCD evolution, and was probably involved in the emergence of a SCD gene expression independent of dietary intakes. Overall, we provide a clear framework of the evolutionary history of SCD genes in vertebrates that should prove instrumental for the functional analysis that may follow.

## Methods

### Sample collection and storage

Adult three-spined stickleback *G. aculeatus *were obtained from the Minho River (North of Portugal) by weir fishing. Adult bichir, *P. senegalus*, were obtained through a local aquarium fish supplier. These freshwater species were kept separately in the lab in dechlorinated tap water within 100 l recirculation systems at appropriate temperature (17°C and 25°C, respectively) and on natural photoperiod. Fish were fed artificial flake or pellet food daily. Adult lesser spotted dogfish, *S.canicula*, were collected by bottom trawling off the northern Portuguese coast. Fish were held in 2000 l seawater tanks in a recirculation system with temperature control (16°C). They were fed mackerel until satiety twice a week.

### Synteny and Paralogy examination

We located the human orthologues of SCD1 (Hsa4) and SCD5 (Hsa10) using the Ensembl genome database release GRCh37. To deduce whether the genomic surroundings of both genes had signs of duplication, attributable to the 2R genome duplications (i.e. part of a paralogon), the gene content in a 1 Mb Ensembl interval was analysed. Gene families with more members mapping to distinct regions were identified. Their phylogeny was determined to infer the duplication pattern. Amino acid sequences were collected from Ensembl and JGI. Gaps and regions of uncertain homology were discarded. The phylogenetic trees were constructed using neighbor-joining from the MEGA4 package [39]. Confidence on each node was assessed by 1000 bootstrap replicates. Synteny data for the examined species was retrieved from the following Ensembl database releases: *H. sapiens *GRCh37, *M. musculus *NCBIM37, *M. domestica *BROADO5, *O. anatinus *OANA5, *G. gallus *WASHUC2, *A. carolinensis *AnoCar1.0, *O. cuniculus *oryCun2, *X. tropicalis *JGI4.1, *G. aculeatus *BROADS1, *T. nigroviridis *TETRAODON8, *T. rubripes *FUGU4, *D. rerio *Zv9, and *O. latipes *MEDAKA1. Mapping information of *B. floridae *SCD's was retrieved from GenBank. We analysed the two immediate ORF's flanking each SCD gene.

### Molecular phylogenetic analysis

SCD1 and SCD5 sequences of *S. canicula *were aligned with those from a wide range of vertebrates and invertebrate species using CLUSTALW in Bioedit. To maximise the number of included species we considered the use of incomplete sequences (e.g. *P. senegalus*). Phylogenetic reconstruction used annotated sequences retrieved from GenBank and Ensembl databases. The accession numbers are given in legend of figure 3. Gaps and regions of uncertain homology were removed to obtain a final alignment of 183 amino acids of 56 sequences. We applied Protest [40] to determine the best model of amino acid substitution (LG+I+G). A Maximum Likelihood tree was constructed with PHYML [41]. The robustness of the tree was assessed through 100 bootstrap replicates of the data. Visualisation was performed in TreeView [42].

### SCD1 and SCD5 isolation in *S. canicula *and *P. senegalus *and gene expression

Total RNA was isolated from the collected tissues (e.g. brain) using the Illustra RNAspin minikit from GE Healthcare (Little Chalfont, UK) with on-column DNase I treatment. The RNA integrity was verified in a 1% agarose gel stained with Gelred (VWR). RNA concentration was measured with a Qubit fluorometer platform (Invitrogen, Carlsbad CA). Conversion of total RNA into first strand cDNA was performed using the iScript cDNA synthesis following the manufacturer recommendations (Bio-Rad). To isolate of SCD1and SCD5 from *S. canicula *and *P. senegalus *we designed degenerate primers with the CODEHOP program [43] (table 1). Various PCR protocols were followed using the Phusion Flash hot start high fidelity polymerase mix with the manufactures recommended conditions (Finnzyme Helsinki FI). Bands of the appropriate size were isolated from the agarose gel (GFX cleaning kit, GE Healthcare), and sequenced directly with one of the flanking primers (Stabvida). Positive sequences were used to design race primers in *S. canicula *with the Primer3 program (v. 0.4.0; [44]). Race cDNA and PCR was prepared according to the SMARTer RACE cDNA kit instructions (Clontech Mountain View CA). Bands were sequenced directly with race primers. Full ORF integrity was determined with flanking primers (table 1). Sequences were deposited in Genbank: ScSCD1: [JF729408], ScSCD5: [JF729409], and PsSCD1: [JF729410].

**Table 1:** List of primers used to isolate and characterize SCD1 and SCD5 genes in *S. canicula *and *P. senegalus*, and expression primers in *S. canicula *and *G. aculeatus.*

Designation	Sequence	Use
SCD5F1deg	5'CACCCCTTCACCTGGHTNTGGGCNTA3'	Isolation of PCR fragment
SCD5R2deg	5' CCAGGCCCAGCCAGMACATRAARTC 3'	Isolation of PCR fragment
SCD5R1deg	5' CGCTCCTTCCGGGCYTSDATCAT 3'	Isolation of PCR fragment
SCD5F2	5'TGTCACGTGGTTGGTGAACAGTGCT3'	Isolation of PCR fragment
SCD5_3Race	5'TCCAGCTGAACCCAACAACATGCTTTA3'	RACE PCR
SCD5_5Race	5' GTTTCCGAAGAAACAGCCAGCCAAT 3'	RACE PCR
SCD5Fexp	5'CCAATTCCATGGCTTTTCAG 3'	gene expression
SCD5Rexp	5'ACACAACAACAGGGTCAGCA3'	gene expression
SCD1F	5'TCGCCAACACCATGGCNTTYCA 3'	Isolation of PCR fragment
SCD1R	5'GAGTAGTCGTAGGGGAAGGTGTGRTGRTARTT 3'	Isolation of PCR fragment
SCD1_3Race	5'GTTGATGTGCTTCATCATCCCCACAGT 3'	RACE PCR
SCD1_5Race	5'ACGAGTTCCAGAGAGTTTCACCCCAGA 3'	RACE PCR
SCD1FSc	5'CTTGCTTAGTGCCCTTGGAG3'	gene expression
SCD1RSc	5' AACGTGCGAGAAGAAAAAGC3'	gene expression
GaSCD1aF	5'GGCCCTATGACAAGAGCATC3'	gene expression
GaSCD1aR	5'CGTAGTCAAAGGGGAACGTG3'	gene expression
GaSCD1bF	5'GATGCTCAACGCCACCTG3'	gene expression
GaSCD1bR	5'AAGGGGAATGTGTGGTGGTA3'	gene expression
SCD1Ffull	5' GGTGTATGCCCGTTCCTTT 3'	ORF PCR
SCD1Rfull	5' TGCGTCAGTGACTCGACAGT 3'	ORF PCR
SCD5Ffull	5' TGGTGAATATGGAGAATCAGGA 3'	ORF PCR
SCD5Rfull	5' TGGACTTTGAGATTTTCTTCGTG 3'	ORF PCR
ScActinf1	5' AGTTGGATGGGTCAGAAAGAC 3'	normalisation
ScActinR1	5' ACGCTCAGTCAGGATCTTCATC 3'	normalisation
GaActinF	5' TCCCTGGAGAAGAGCTACGA 3'	normalisation
GaActinR	5' GTGTTGGCGTACAGGTCCTT 3'	normalisation

Gene expression was verified through RT-PCR with specific primers, which were tentatively designed to outflank conserved introns (Table 1). Briefly, total RNA from the various tissue samples was converted into cDNA as described above. The same concentration input per sample was used. One microliter of cDNA was used in a PCR reaction with the following cycle conditions: 94°C 30 sec, 55°C 30 sec, 72°C 30 sec for 30 cycles (to avoid the PCR plateau). Samples were run on a 2% agarose gel, stained with Gelred, and digitized images acquired for analysis (LAS4000mini, FujiFilm, Tokyo, Japan). Actin was used to normalize gene expression [45].

## Authors' contributions

The original idea for this study was conceived by LFCC and IC. LFCC performed all the experimental analysis; JMW, OG and SGO prepared all the biological samples up to the use in PCR used in the study. LFCC, ER, and IC participated in the discussion regarding lipid metabolism and physiology. The manuscript was written by LFCC, and edited by all other co-authors. All authors have read and approved the final manuscript.

## Supplementary Material

Additional file 1 **Evolutionary relationships of WNT8 (A), HNRNP (B), CHUK (C), ERLIN (D), SEC31 (E), PLAC8 (F), and TMEM150 (G)**. Click here for file

Additional file 2 **Gene expression of SCD1a and SCD1b in *G. aculeatus *tissues**. E - eye, B-brain, H-heart, Sp-spleen, L-liver, O-ovary, Sk-skin, G-gill, St-stomach, Hg-hind gut. Click here for file

## References

[B1] Shanklin J Cahoon EB Desaturation and related modifications of fatty acids Ann Rev Plant Mol Biol 1998 49 611 641 10.1146/annurev.arplant.49.1.611 15012248

[B2] Enoch HG Catalá A Strittmatter P Mechanism of rat liver microsomal stearyl-CoA desaturase. Studies of the substrate specificity, enzyme-substrate interactions, and the function of lipid J Biol Chem 1976 251 16 5095 103 8453

[B3] Flowers MT Ntambi JM Role of stearoyl-coenzyme A desaturase in regulating lipid metabolism Curr Opin Lipidol 2008 19 3 248 56 10.1097/MOL.0b013e3282f9b54d 18460915 PMC4201499

[B4] Miyazaki M Ntambi JM Role of stearoyl-coenzyme A desaturase in lipid metabolism Prostaglandins Leukot Essent Fatty Acids 2003 68 2 113 21 10.1016/S0952-3278(02)00261-2 12538075

[B5] Ntambi JM The regulation of stearoyl-CoA desaturase (SCD) Prog Lipid Res 1995 34 2 139 50 10.1016/0163-7827(94)00010-J 7480063

[B6] Ntambi JM Buhrow SA Kaestner KH Christy RJ Sibley E Kelly TJ Jr Lane MD Differentiation-induced gene expression in 3T3-L1 preadipocytes. Characterization of a differentially expressed gene encoding stearoyl-CoA desaturase J Biol Chem 1988 263 33 17291 300 2903162

[B7] Jeffcoat R James AT The control of stearoyl-CoA desaturase by dietary linoleic acid FEBS Lett 1978 85 1 114 8 10.1016/0014-5793(78)81260-5 23314

[B8] Price PT Nelson CM Clarke SD Omega-3 polyunsaturated fatty acid regulation of gene expression Curr Opin Lipidol 2000 11 1 3 7 10.1097/00041433-200002000-00002 10750688

[B9] Ntambi JM Bené H Polyunsaturated fatty acid regulation of gene expression J Mol Neurosci 2001 16 2-3 273 8 discussion 279-84 10.1385/JMN:16:2-3:273 11478382

[B10] Ntambi JM Miyazaki M Stoehr JP Lan H Kendziorski CM Yandell BS Song Y Cohen P Friedman JM Attie AD Loss of stearoyl-CoA desaturase-1 function protects mice against adiposity Proc Natl Acad Sci USA 2002 99 17 11482 6 10.1073/pnas.132384699 12177411 PMC123282

[B11] Miyazaki M Kim YC Gray-Keller MP Attie AD Ntambi JM The biosynthesis of hepatic cholesterol esters and triglycerides is impaired in mice with a disruption of the gene for stearoyl-CoA desaturase 1 J Biol Chem 2000 275 39 30132 8 10899171 10.1074/jbc.M005488200

[B12] Hsieh SL Hu CY Hsu YT Hsieh TJ Influence of dietary lipids on the fatty acid composition and stearoyl-CoA desaturase expression in hybrid tilapia (Oreochromis niloticusxO. aureus) under cold shock Comp Biochem Physiol B Biochem Mol Biol 2007 147 3 438 44 10.1016/j.cbpb.2007.02.010 17409004

[B13] Evans H De Tomaso T Quail M Rogers J Gracey AY Cossins AR Berenbrink M Ancient and modern duplication events and the evolution of stearoyl-CoA desaturases in teleost fishes Physiol Genomics 2008 35 1 18 29 10.1152/physiolgenomics.90266.2008 18593860 PMC2536826

[B14] Polley SD Tiku PE Trueman RT Caddick MX Morozov IY Cossins AR Differential expression of cold- and diet-specific genes encoding two carp liver delta 9-acyl-CoA desaturase isoforms Am J Physiol Regul Integr Comp Physiol 2003 284 1 R41 50 12388470 10.1152/ajpregu.00263.2002

[B15] Mauvoisin D Mounier C Hormonal and nutritional regulation of SCD1 gene expression Biochimie 2010 in press 10.1016/j.biochi.2010.08.001 20713121

[B16] Miyazaki M Jacobson MJ Man WC Cohen P Asilmaz E Friedman JM Ntambi JM Identification and characterization of murine SCD4, a novel heart-specific stearoyl-CoA desaturase isoform regulated by leptin and dietary factors J Biol Chem 2003 278 36 33904 11 10.1074/jbc.M304724200 12815040

[B17] Wang J Yu L Wang H Gao Y Schrementi JP Porter RK Yurek DA Kuo M Suen CS Cao G Bean JS Kauffman RF Qian Y Identification and characterization of hamster stearoyl-CoA desaturase isoforms Lipids 2008 43 3 197 205 10.1007/s11745-007-3139-0 18084785

[B18] Wang J Yu L Schmidt RE Su C Huang X Gould K Cao G Characterization of HSCD5, a novel human stearoyl-CoA desaturase unique to primates Biochem Biophys Res Commun 2005 332 3 735 42 10.1016/j.bbrc.2005.05.013 15907797

[B19] Lengi AJ Corl BA Identification and characterization of a novel bovine stearoyl-CoA desaturase isoform with homology to human SCD5 Lipids 2007 42 6 499 508 10.1007/s11745-007-3056-2 17468887

[B20] Lengi AJ Corl BA Comparison of pig, sheep and chicken SCD5 homologs: Evidence for an early gene duplication event Comp Biochem Physiol B Biochem Mol Biol 2008 150 4 440 6 10.1016/j.cbpb.2008.05.001 18556229

[B21] Putnam NH Butts T Ferrier DE Furlong RF Hellsten U Kawashima T Robinson-Rechavi M Shoguchi E Terry A Yu JK Benito-Gutiérrez EL Dubchak I Garcia-Fernàndez J Gibson-Brown JJ Grigoriev IV Horton AC de Jong PJ Jurka J Kapitonov VV Kohara Y Kuroki Y Lindquist E Lucas S Osoegawa K Pennacchio LA Salamov AA Satou Y Sauka-Spengler T Schmutz J Shin-I T Toyoda A Bronner-Fraser M Fujiyama A Holland LZ Holland PW Satoh N Rokhsar DS The amphioxus genome and the evolution of the chordate karyotype Nature 2008 453 7198 1064 71 10.1038/nature06967 18563158

[B22] Jaillon O Aury JM Brunet F Petit JL Stange-Thomann N Mauceli E Bouneau L Fischer C Ozouf-Costaz C Bernot A Nicaud S Jaffe D Fisher S Lutfalla G Dossat C Segurens B Dasilva C Salanoubat M Levy M Boudet N Castellano S Anthouard V Jubin C Castelli V Katinka M Vacherie B Biémont C Skalli Z Cattolico L Poulain J De Berardinis V Cruaud C Duprat S Brottier P Coutanceau JP Gouzy J Parra G Lardier G Chapple C McKernan KJ McEwan P Bosak S Kellis M Volff JN Guigó R Zody MC Mesirov J Lindblad-Toh K Birren B Nusbaum C Kahn D Robinson-Rechavi M Laudet V Schachter V Quétier F Saurin W Scarpelli C Wincker P Lander ES Weissenbach J Roest Crollius H Genome duplication in the teleost fish Tetraodon nigroviridis reveals the early vertebrate proto-karyotype Nature 2004 431 7011 946 57 10.1038/nature03025 15496914

[B23] Pollard SL Holland PW Evidence for 14 homeobox gene clusters in human genome ancestry Curr Biol 2000 10 17 1059 62 10.1016/S0960-9822(00)00676-X 10996074

[B24] Luke GN Castro LF McLay K Bird C Coulson A Holland PW Dispersal of NK homeobox gene clusters in amphioxus and humans Proc Natl Acad Sci USA 2003 100 9 5292 5 10.1073/pnas.0836141100 12704239 PMC154338

[B25] Springer MS Murphy WJ Eizirik E O'Brien SJ Placental mammal diversification and the Cretaceous-Tertiary boundary Proc Natl Acad Sci USA 2003 100 3 1056 61 10.1073/pnas.0334222100 12552136 PMC298725

[B26] Takeuchi M Okabe M Aizawa S The genus Polypterus (bichirs): a fish group diverged at the stem of ray-finned fishes (Actinopterygii) Cold Spring Harb Protoc 2009 2009 5 pdb.emo117 10.1101/pdb.emo117 20147149

[B27] Venkatesh B Kirkness EF Loh YH Halpern AL Lee AP Johnson J Dandona N Viswanathan LD Tay A Venter JC Strausberg RL Brenner S Survey sequencing and comparative analysis of the elephant shark (Callorhinchus milii) genome PLoS Biol 2007 5 4 e101 10.1371/journal.pbio.0050101 17407382 PMC1845163

[B28] Zheng Y Prouty SM Harmon A Sundberg JP Stenn KS Parimoo S Scd3--a novel gene of the stearoyl-CoA desaturase family with restricted expression in skin Genomics 2001 71 2 182 91 10.1006/geno.2000.6429 11161812

[B29] Furlong RF Graham A Vertebrate neurogenin evolution: long-term maintenance of redundant duplicates Dev Genes Evol 2005 215 12 639 44 10.1007/s00427-005-0023-x 16220265

[B30] Mulley JF Chiu CH Holland PW Breakup of a homeobox cluster after genome duplication in teleosts Proc Natl Acad Sci USA 2006 103 27 10369 72 10.1073/pnas.0600341103 16801555 PMC1502464

[B31] Flores MV Hall CJ Davidson AJ Singh PP Mahagaonkar AA Zon LI Crosier KE Crosier PS Intestinal differentiation in zebrafish requires Cdx1b, a functional equivalent of mammalian Cdx2 Gastroenterology 2008 135 5 1665 75 10.1053/j.gastro.2008.07.024 18804112

[B32] Morais S Monroig O Zheng X Leaver MJ Tocher DR Highly unsaturated fatty acid synthesis in Atlantic salmon: characterization of ELOVL5- and ELOVL2-like elongases Mar Biotechnol (NY) 2009 11 5 627 39 10.1007/s10126-009-9179-0 19184219

[B33] Chang BE Hsieh SL Kuo CM Molecular cloning of full-length cDNA encoding delta-9 desaturase through PCR strategies and its genomic organization and expression in grass carp (Ctenopharyngodon idella) Mol Reprod Dev 2001 58 3 245 54 10.1002/1098-2795(200103)58:3<245::AID-MRD1>3.0.CO;2-7 11170264

[B34] Garbay B Boiron-Sargueil F Shy M Chbihi T Jiang H Kamholz J Cassagne C Regulation of oleoyl-CoA synthesis in the peripheral nervous system: demonstration of a link with myelin synthesis J Neurochem 1998 71 4 1719 26 9751207 10.1046/j.1471-4159.1998.71041719.x

[B35] Bourre JM Dumont OL Clément ME Durand GA Endogenous synthesis cannot compensate for absence of dietary oleic acid in rats J Nutr 1997 127 3 488 93 9082035 10.1093/jn/127.3.488

[B36] Edmond J Higa TA Korsak RA Bergner EA Lee WN Fatty acid transport and utilization for the developing brain J Neurochem 1998 70 3 1227 34 9489745 10.1046/j.1471-4159.1998.70031227.x

[B37] Cnop M Fatty acids and glucolipotoxicity in the pathogenesis of Type 2 diabetes Biochem Soc Trans 2008 36 Pt 3 348 52 18481955 10.1042/BST0360348

[B38] Hellemans KH Hannaert JC Denys B Steffensen KR Raemdonck C Martens GA Van Veldhoven PP Gustafsson JA Pipeleers D Susceptibility of pancreatic beta cells to fatty acids is regulated by LXR/PPARalpha-dependent stearoyl-coenzyme A desaturase PLoS One 2009 4 9 e7266 10.1371/journal.pone.0007266 19787047 PMC2746288

[B39] Tamura K Dudley J Nei M Kumar S MEGA4: Molecular Evolutionary Genetics Analysis (MEGA) software version 4.0 Molecular Biology and Evolution 2007 24 1596 1599 10.1093/molbev/msm092 17488738

[B40] Abascal F Zardoya R Posada D ProtTest: Selection of best-fit models of protein evolution Bioinformatics 2005 21 9 2104 2105 10.1093/bioinformatics/bti263 15647292

[B41] Guindon S Gascuel O A simple, fast, and accurate algorithm to estimate large phylogenies by maximum likelihood Systematic Biology 2003 52 5 696 704 10.1080/10635150390235520 14530136

[B42] Page RDM TREEVIEW: An application to display phylogenetic trees on personal computers Computer Applications in the Biosciences 1996 12 357 358 8902363 10.1093/bioinformatics/12.4.357

[B43] Rose TM Schultz ER Henikoff JG Pietrokovski S McCallum CM Henikoff S Consensus-degenerate hybrid oligonucleotide primers for amplification of distantly related sequences Nucleic Acids Res 1998 26 7 1628 35 10.1093/nar/26.7.1628 9512532 PMC147464

[B44] Rozen S Skaletsky HJ Krawetz S, Misener S Primer3 on the www for general users and for biologist programmers Bioinformatics Methods and Protocols: Methods in Molecular Biology 2000 Humana Press, Totowa, NJ 365 386 10.1385/1-59259-192-2:365 10547847

[B45] Mulley JF Holland PW Parallel retention of Pdx2 genes in cartilaginous fish and coelacanths Mol Biol Evol 2010 27 10 2386 91 10.1093/molbev/msq121 20463047 PMC2944030

