# Supplementary material for: The evolutionary history of the stearoyl-CoA desaturase gene family in vertebrates
Source: BMC Evol Biol. 2011 May 19;11:132. doi: 10.1186/1471-2148-11-132 (PMC3112091; doi:10.1186/1471-2148-11-132)
Supplement: Additional file 1 — Evolutionary relationships of WNT8 (A), HNRNP (B), CHUK (C), ERLIN (D), SEC31 (E), PLAC8 (F), and TMEM150 (G). [file 1471-2148-11-132-S1.DOC]

**(A)**

GaWNT8Ab ENSGACP00000024426

OlWNT8Ab ENSORLP00000009584

DrWNT8Ab ENSDARP00000096426

DrWNT8Aa ENSDARP00000116057

GaWNT8Aa ENSGACP00000024432

OlWNT8Aa ENSORLP00000009605

GgWNT8A ENSGALP00000009803

AcWNT8A ENSACAP00000016713

XtWNT8A ENSXETP00000008323

HsWNT8A ENSP00000381739

MmWNT8A ENSMUSP00000012426

GaWNT8B ENSGACP00000011213

OlWNT8B ENSORLP00000008607

DrWNT8B ENSDARP00000049623

XtWNT8B ENSXETP00000051242

GgWNT8B ENSGALP00000039341

HsWNT8B ENSP00000340677

MmWNT8B ENSMUSP00000042867

BfWNT8 AF190470

Cs ENSCSAVP00000008613

100

100

100

99

100

99

99

54

44

100

100

81

53

91

76

86

49

0.1

**(B)**

GaHNRNPABb ENSGACP00000027539

OlHNRNPABb ENSORLP00000019411

DrHNRNPABb ENSDARP00000089179

DrHNRNPABa ENSDARP00000013795

GaHNRNPABa ENSGACP00000023937

OlHNRNPABa ENSORLP00000006716

XtHNRNPAB ENSXETP00000008339

AcHNRNPAB ENSACAP00000016687

HsHNRNPAB ENSP00000351108

MmHNRNPAB ENSMUSP00000104731

GaHNRNPD ENSGACP00000020678

DrHNRNPD ENSDARP00000092188

OlHNRNPD ENSORLP00000002282

XtHNRNPD ENSXETP00000027494

AcHNRNPD ENSACAP00000010205

HsHNRNPD ENSP00000313199

MmHNRNPD ENSMUSP00000132735

DrHNRPDL ENSDARP00000005968

XtHNRPDL ENSXETP00000027520

GgHNRPDL ENSGALP00000037222

HsHNRPDL ENSP00000295470

MmHNRPDL ENSMUSP00000121005

Cs1 ENSCSAVP00000000338

Cs2 ENSCSAVP00000016637

Ci ENSCINP00000007991

100

90

99

96

100

100

100

90

62

91

72

100

99

100

99

67

94

86

93

63

70

42

0.05

**(C)**

HsCHUK ENSP00000359424

MmCHUK ENSMUSP00000026217

GgCHUK ENSGALP00000005196

AcCHUK ENSACAP00000011973

XtCHUK ENSXETP00000038296

GaCHUKa ENSGACP00000008884

OlCHUKa ENSORLP00000011761

DrCHUKb ENSDARP00000013115

GaCHUKb ENSGACP00000005520

OlCHUKb ENSORLP00000016017

GaIKBKB ENSGACP00000005440

OlIKBKB ENSORLP00000003067

DrIKBKB ENSDARP00000117685

XtIKBKB ENSXETP00000036619

HsIKBKB ENSP00000430684

MmIKBKB ENSMUSP00000033939

GgIKBKB ENSGALP00000005924

AcIKBKB ENSACAP00000012680

Bf XP_002604730

Cg AAC05683

100

100

100

94

99

100

100

100

100

100

99

100

100

100

100

99

73

0.1

**(D)**

HsERLIN1 ENSP00000410964

MmERLIN1 ENSMUSP00000107659

GgERLIN1 ENSGALP00000005232

XtERLIN1 ENSXETP00000001887

DrERLIN1 ENSDARP00000015815

GaERLIN1 ENSGACP00000008924

OlERLIN1 ENSORLP00000011710

GaERLIN2 ENSGACP00000017558

OlERLIN2 ENSORLP00000019471

XtERLIN2 ENSXETP00000052598

HsERLIN2 ENSP00000276461

MmERLIN2 ENSMUSP00000033873

GgERLIN2 ENSGALP00000004879

AcERLIN2 ENSACAP00000000170

NvERLIN XP_001622641

CiERLIN ENSCINP00000008668

CsERLIN ENSCSAVP00000018861

100

100

100

100

75

100

93

99

94

68

93

100

60

0.05

54

**(E)**

HsSEC31B ENSP00000359370

MmSEC31B ENSMUSP00000064900

GgSEC31B ENSGALP00000039343

XtSEC31B ENSXETP00000031054

DrSEC31B ENSDARP00000008006

GaSEC31B ENSGACP00000003156

OlSEC31B ENSORLP00000011637

HsSEC31A ENSP00000400926

MmSEC31A ENSMUSP00000092157

GgSEC31A ENSGALP00000018179

DrSEC31A ENSDARP00000104171

GaSEC31A ENSGACP00000020706

OlSEC31A ENSORLP00000002335

CiSEC31 ENSCINP00000022054

CsSEC31 ENSCSAVP00000016511

BfSEC31 XP_002602930

100

100

100

100

100

100

100

100

100

93

100

100

90

0.05

**(F)**

**GaTMEM150A** ENSGACP00000016916

**OlTMEM150A** ENSORLP00000018660

**DrTMEM150A** ENSDARP00000089316

**HsTMEM150A** ENSP00000334708

**AcTMEM150A** ENSACAP00000017777

**XtTMEM150A** ENSXETP00000002721

**GgTMEM150D** ENSGALP00000009048

**AcTMEM150D** ENSACAP00000014181

**GaTMEM150Db E**NSGACP00000005552

**GaTMEM150Da** ENSGACP00000010525

**TnTMEM150Da** ENSTNIP00000004522

**DrTMEM150Db**

**HsTMEM150C E**NSP00000403438

**GgTMEM150C** ENSGALP00000018196

**DrTMEM150C** ENSDARP00000092177

**TrTMEM150C** ENSTRUP00000047222

**GaTMEM150B** ENSGACP00000010976

**DrTMEM150B** ENSDARP00000073041

**XtTMEM150B** ENSXETP00000054331

**HsTMEM150B** ENSP00000320757

**MmTMEM150C** ENSMUSP00000083549

**Ci** ENSCINP00000008319

100

100

100

100

99

94

89

99

99

100

100

74

74

73

85

100

93

0.2

**Figure** Evolutionary relationships of WNT8 (A), HNRNP (B), CHUK (C), ERLIN (D), SEC31 (E), and TMEM150 (F).The evolutionary history was inferred using the Neighbor-Joining method. The percentage of replicate trees in which the associated taxa clustered together in the bootstrap test (100 replicates) are shown next to the branches. Species names as follows: Ci- *Ciona intestinalis*, Cs- *Ciona savignyi*, Bf-*Branchiostoma floridae*, Ac- *A. carolinensis*, Hs- *H. sapiens*, Mm- *M. musculus*, Gg- *G. gallus*, Xt- *X. tropicalis*, Ga- *G. aculeatus*, Ol- *O. latipes*, Tr- *T. rubripes*, Tn- *T. nigroviridis*, Nv- *Nematostella vectensis*, Cg- *Crassostrea gigas*. Ensembl numbers and GenBank accession numbers are given in front of each clade.
