# Supplementary figures and images for: The evolutionary history of the stearoyl-CoA desaturase gene family in vertebrates
Source: BMC Evol Biol. 2011 May 19;11:132. doi: 10.1186/1471-2148-11-132 (PMC3112091; doi:10.1186/1471-2148-11-132)

E B H Sp L O Sk G St Hg -

200bp

100bp

**GaSCD1a**

200bp

100bp

**GaSCD1b**

200bp

100bp

**GaActin**

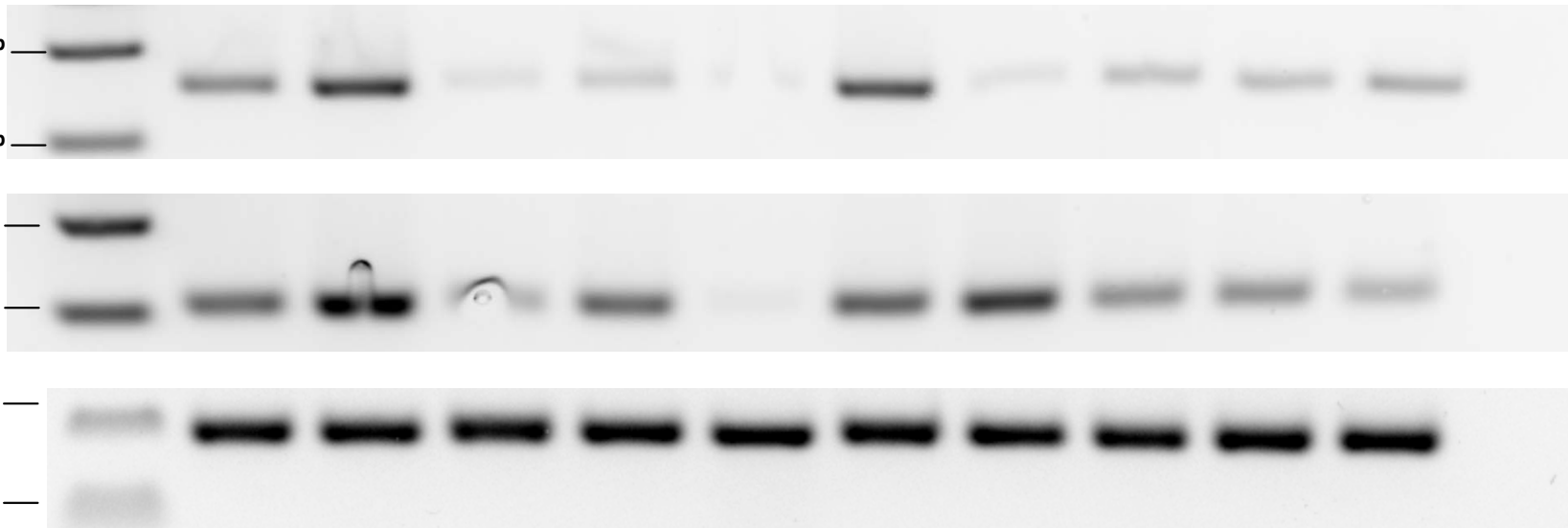

Supplement: Additional file 2 — Gene expression of SCD1a and SCD1b in G. aculeatus tissues. E - eye, B-brain, H-heart, Sp-spleen, L-liver, O-ovary, Sk-skin, G-gill, St-stomach, Hg-hind gut. [file 1471-2148-11-132-S2.PDF]
